# Al_2_O_3_/HfO_2_ Nanolaminate Dielectric Boosting IGZO-Based Flexible Thin-Film Transistors

**DOI:** 10.1007/s40820-022-00929-y

**Published:** 2022-09-27

**Authors:** Qiuwei Shi, Izzat Aziz, Jin-Hao Ciou, Jiangxin Wang, Dace Gao, Jiaqing Xiong, Pooi See Lee

**Affiliations:** 1 School of Materials Science and Engineering, Nanyang Technological University, 50 Nanyang Avenue, Singapore, 639798 Singapore; 2 School of Chemistry and Materials Science, Nanjing University of Information Science and Technology, Nanjing, 210044 People’s Republic of China

**Keywords:** Nanolaminate dielectric, Al_2_O_3_/HfO_2_, Thin-film transistors, Flexible electronic

## Abstract

**Highlights:**

- A stable laminated Al_2_O_3_/HfO_2_ insulator is developed by atomic layer deposition at a relatively lower temperature of 150 °C.
- The flexible thin-film transistors (TFTs) with bottom-gate top-contacted configuration are fabricated on a flexible substrate with the Al_2_O_3_/HfO_2_ insulator.
- The flexible TFTs present the carrier mobilities of 9.7 cm^2^ V^−1^ s^−1^, ON/OFF ratio of ~ 1.3 × 10^6^, subthreshold voltage of 0.1 V, saturated current up to 0.83 mA, and subthreshold swing of 0.256 V dec^−1^.

**Abstract:**

Flexible thin-film transistors (TFTs) have attracted wide interest in the development of flexible and wearable displays or sensors. However, the conventional high processing temperatures hinder the preparation of stable and reliable dielectric materials on flexible substrates. Here, we develop a stable laminated Al_2_O_3_/HfO_2_ insulator by atomic layer deposition at a relatively lower temperature of 150 °C. A sputtered amorphous indium-gallium-zinc oxide (IGZO) with the stoichiometry of In_0.37_Ga_0.20_Zn_0.18_O_0.25_ is used as the active channel material. The flexible TFTs with bottom-gate top-contacted configuration are further fabricated on a flexible polyimide substrate with the Al_2_O_3_/HfO_2_ nanolaminates. Benefited from the unique structural and compositional configuration in the nanolaminates consisting of amorphous Al_2_O_3_, crystallized HfO_2_, and the aluminate Al–Hf–O phase, the as-prepared TFTs present the carrier mobilities of 9.7 cm^2^ V^−1^ s^−1^, ON/OFF ratio of ~ 1.3 × 10^6^, subthreshold voltage of 0.1 V, saturated current up to 0.83 mA, and subthreshold swing of 0.256 V dec^−1^, signifying a high-performance flexible TFT, which simultaneously able to withstand the bending radius of 40 mm. The TFTs with nanolaminate insulator possess satisfactory humidity stability and hysteresis behavior in a relative humidity of 60–70%, a temperature of 25–30 °C environment. The yield of IGZO-based TFTs with the nanolaminate insulator reaches 95%.
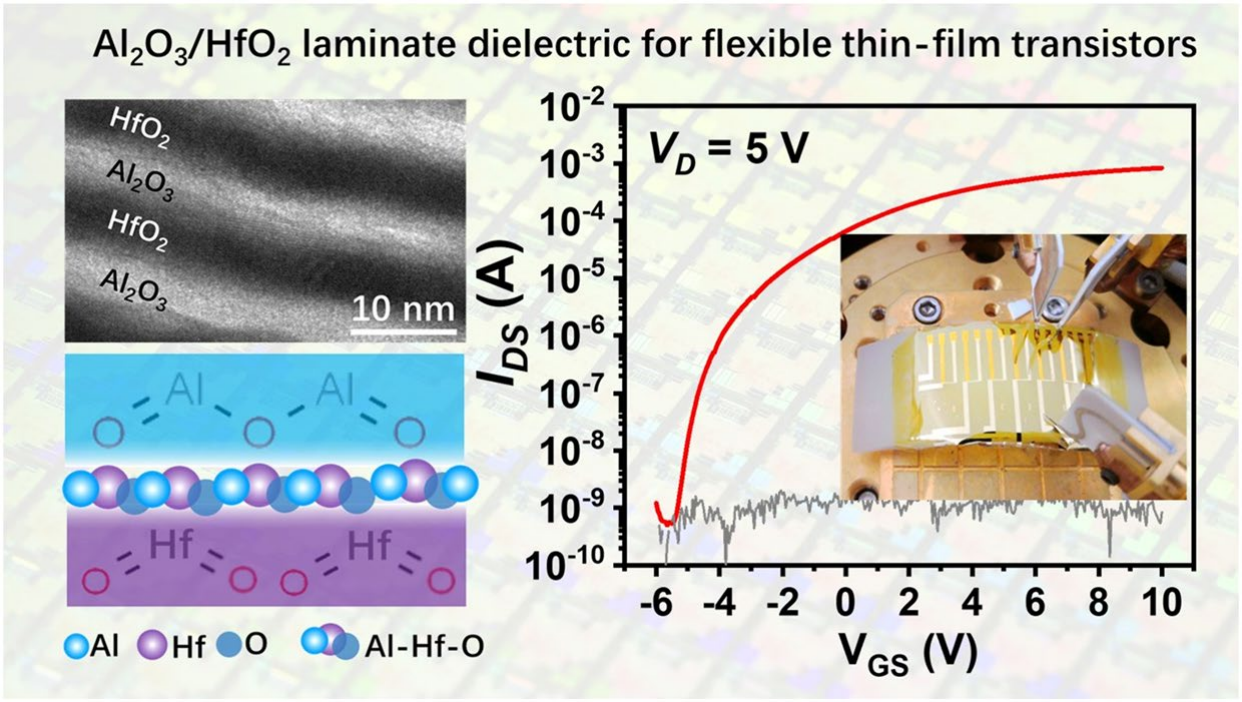

**Supplementary Information:**

The online version contains supplementary material available at 10.1007/s40820-022-00929-y.

## Introduction

The development and application of microelectronics have brought us to the era of digital and information age. Nowadays, thin-film transistors (TFTs) with the active channel layer of IGZO [1, 2], In_2_O_3_-ZnO, ZnO, and SnO_2_ are the most basic and important component in modern electronic devices and equipments [3, 4], which have been widely used in sensors [5, 6], switching memories [7], logical circuits, and especially in flat-panel displays [8, 9]. The highly integrated portable electronic devices need high-performance TFTs, for example, in active matrix display for touch panels, flexible circuitries in smartphone, smartwatch, and laptop consumers products with features such as high brightness, high screen resolution and refresh rate, foldable, and low energy consumption. Thus, the great demand for flexible, high-performance, and high energy efficiency TFTs has emerged [10]. Recently, flexible electronics attracted great interest in the field of wearable health management devices and flexible displays for their intrinsic properties including bendable, deformable, and portable [11, 12]. Especially, as essential components of the pixel-drive circuits, TFTs work as the driving switches playing a significant role in organic light-emitting displays and liquid–crystal displays [9]. Accordingly, great efforts (such as the deep subthreshold regime operatable schottky-barrier IGZO thin-film transistor) are being conducted to develop low power, high current sensitivity, and flexible TFTs [13]. These high-performance flexible TFTs could be used as the key units for future displays, artificial skin, and soft robotics [14–17].

To date, combinations of advanced materials to fabricate flexible TFTs with enhanced intrinsic electrical properties and ingenious device structures with optimized configurations have been studied and explored. Despite many great progresses such as 1D carbon nanotubes (CNTs) or nanowire flexible TFTs [18–22], 2D nanomaterials flexible TFTs [23, 24], flexible organic TFTs [25–28], and metal-oxide flexible TFTs [29–31] have reported to obtain flexible TFTs. The developed flexible TFTs are still difficult to meet the practical application demands. This is due to the considerably the high standard and stringent requirements of electronic products ranging from superior electrical output performance, continuous/scale-up fabrication, reliability, and environmental stability. For CNTs flexible TFTs, although the single CNTs TFTs have been demonstrated with superior performance [32], but these is not suitable for assembling large-scale integrated circuits due to limiting factors such as cost, uniformity, and purity of network-type CNTs [19]. Regarding 2D materials TFTs, a wide variety of 2D nanosheets including boron nitride dielectric, graphene, MoS_2_, and WS_2_ channels can be used to prepare flexible TFTs with high mobilities [33], yet optimizing the flake sizes and continuous reliable operability are still needed to be explored. The organic semiconductors with intrinsic mechanical properties provide a promising way towards fabricating flexible TFTs. However, challenges such as environment robustness, shelf-life, and device performance are still needed to be circumvented. Comparing with the organic TFTs, metal-oxide flexible TFTs are compatible with conventional complementary metal–oxide–semiconductor processes and offer higher performance and stability [1, 34, 35], but typically need a relatively high-temperature annealing process [13, 36]. Usually, the polymeric substrates with relatively low glass transition temperature (*T*_g_) such as polyethylene terephthalate (PET), polyethylene naphthalate (PEN), and polyimide (PI) for flexible TFTs are easily degraded during high-temperature processing [9, 31, 37]. Besides, the thermal explosion coefficient difference between layer for flexible TFTs will result in the internal stresses, decreasing the device performance and stability. Therefore, exploring flexible TFTs at low temperature (200 °C or lower) with ideal device performance is of considerable importance for flexible electronics.

In this work, high-performance flexible TFTs with bottom-gate top-contacted configuration were successfully fabricated on a polyimide substrate without any post-annealing process. The amorphous indium-gallium-zinc oxide (a-IGZO, with the stoichiometry of In_0.37_Ga_0.20_Zn_0.18_O_0.25_), was deposited by radio frequency (RF) sputtering and used as the active channel. The Al_2_O_3_/HfO_2_ laminated insulator was prepared by atomic layer deposition (ALD) at a relatively lower temperature of 150 °C. Benefited from the nanolaminates of Al_2_O_3_/HfO_2_ dielectric consisting of amorphous Al_2_O_3_ and crystallize HfO_2_, and the interface aluminate Al–Hf–O phase, the as-prepared TFTs exhibited the carrier mobility of 9.7 cm^2^ V^−1^ s^−1^, ON/OFF ratio ~ 1.3 × 10^6^, subthreshold voltage of 0.1 V, saturated current up to 0.70 mA, and subthreshold swing of 0.256 V dec^−1^, as well as withstanding the bending radius of 40 mm. Furthermore, the TFTs with nanolaminate insulator possess satisfactory humidity stability and hysteresis behavior in a relative humidity of 60–70%, a temperature of 25–30 °C environment. The yield of IGZO-based TFTs with the nanolaminate insulator reaches 95%.

## Experimental

### Device Fabrication

Two types of substrates including the rigid Si wafers and flexible polyimide films were used for the TFTs fabrication. The detailed fabrication procedure for polyimide (PI) substrate-based flexible TFTs will be introduced below. First, the poly(pyromellitic dianhydride-co-4,4’-oxydianiline), amic acid (PAA) solution precursor (purchased from Sigma Aldrich) was spun coated onto the SiO_2_/Si wafer at the spin rate of 1000 rpm for 1 min. Then, the PAA-coated wafer was moving into a tube furnace for annealing at 300 °C for 1 h with Ar flow for complete curing and imidization. The as-obtained PI film on the wafer was about 15 μm thick. Subsequently, the Ti/Au gate electrode with the thickness of 10/100 nm was deposited through an e-beam evaporator. A series of the thicker dielectric layers containing more layers of Al_2_O_3_/HfO_2_ nanolaminate dielectric was used to fabricate the flexible TFTs. After a series of qualifications, the thickness of the nanolaminate dielectric was finally set at 20 nm. The nanolaminate Al_2_O_3_/HfO_2_ (five layers composing of three layers of Al_2_O_3_ and two layers of HfO_2_, the thickness of each layer is 4 nm) insulator was deposited using the Cambridge Nanotech ALD equipment using H_2_O as the oxygen source, applying trimethylaluminum (TMA) and tetrakis(dimethylamido) hafnium (TDMAH) as their metal sources. Prior to deposition processing, the TDMAH was heated to 75 °C, while TMA and H_2_O were kept at room temperature. The pulses sequence of one deposition cycle to obtain 0.1 nm Al_2_O_3_ was as follows: TMA exposure (0.25 s), N_2_ purging (5 s), H_2_O exposure (0.25 s), N_2_ purging (5 s). The pulses sequence for obtaining 0.1 nm HfO_2_ was as follows: TDMAH exposure (0.5 s), N_2_ purging (5 s), H_2_O exposure (0.1 s), N_2_ purging (5 s). Afterward, a 50-nm-thick patterned IGZO channel layer was deposited at room temperature using the Denton Sputtering System. The process conditions applied for IGZO deposition include the pre-vacuum of 10^−6^ Torr, O_2_ and Ar rate of 5 and 50 sccm, respectively, the RF sputtering power of 100 W. We have identified the structure and stoichiometry of the active layer (IGZO) with FIB-TEM and EDX. The red wireframe part in Fig. S1a shows that the IGZO active layer was in an amorphous state. The In/Ga/Zn/O atomic content percentage in IGZO was demonstrated as In_0.37_Ga_0.20_Zn_0.18_O_0.25_ (Fig. S1 and S2). Lastly, the patterned Ti/Au source and drain electrodes with the thickness of 10/100 nm were further deposited by E-beam evaporation, followed by lift-off to obtain the flexible TFTs for measurements. The channel length and width of TFT devices are 20 and 100 μm, respectively.

### Film and Device Characterization

The microscope images were taken by the Olympus SZX16 optical microscope. All TFTs characterizations were performed using a Keithley 4200 semiconductor analyzer in ambient environment. The capacitances of the as-prepared insulators in metal–insulator–metal structures were measured by the Keysight (Agilent) LCR meter. The cross-sectional sample for transmission electron microscope (TEM) and energy-dispersive X-ray spectroscopy (EDS) measurements were prepared by a dual-beam FIB-SEM system (Zeiss Crossbeam 540). The TEM/EDS characterizations were carried out via the JEOL transmission electron microscope system at (JEM-2100 UHR at 200 kV and JED 2300 T EDS). X-ray photoelectron spectroscopy (XPS) of the as-prepared insulators was characterized with the PHI Quantera II surface analysis equipment. All the obtained XPS spectra were calibrated by the adsorbed C 1*s* (284.6 eV).

## Results and Discussion

**Fig. 1:**
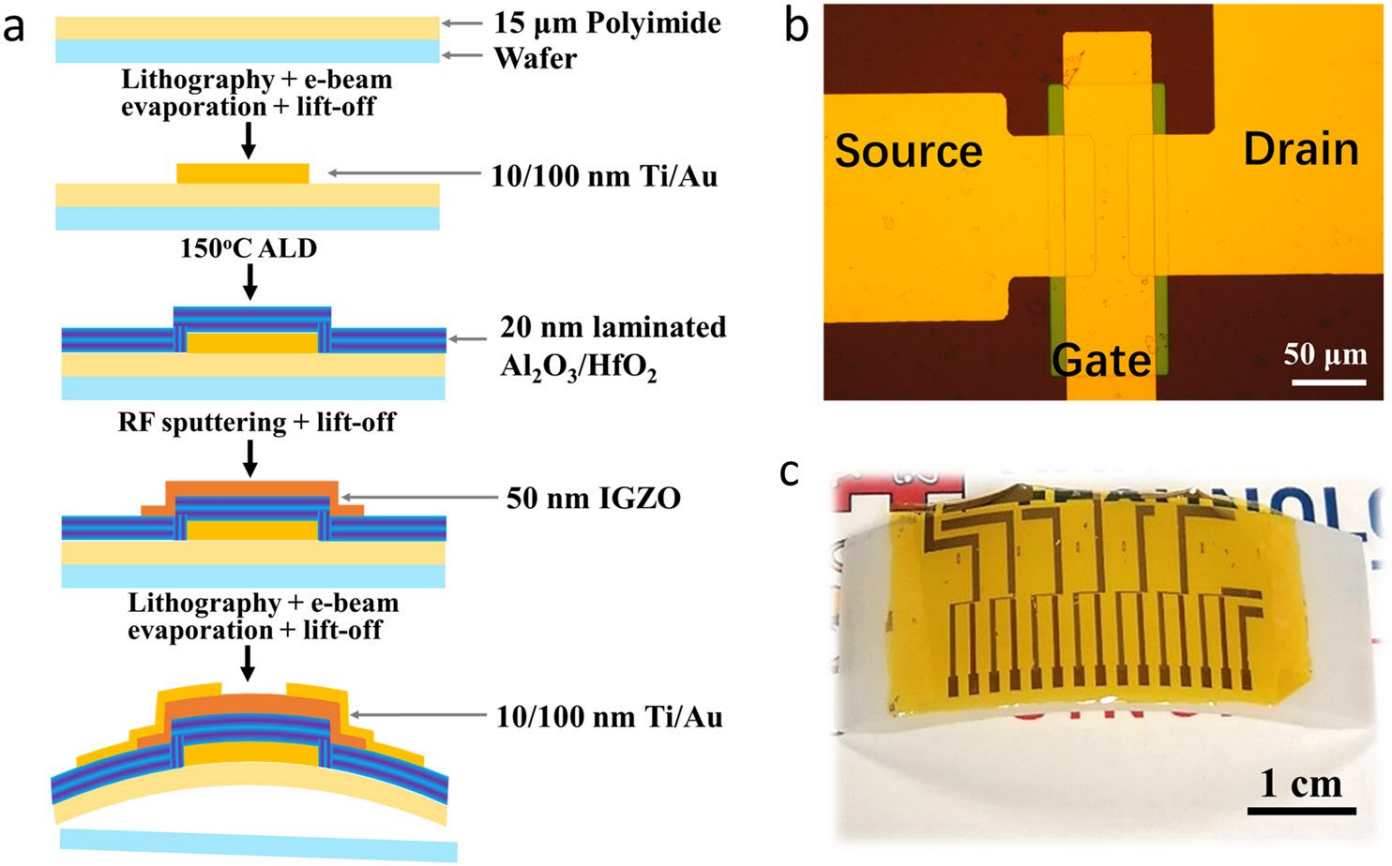
**a** Schematic diagram of the fabrication flow of the IGZO-based TFT with laminated Al_2_O_3_/HfO_2_ insulator including the respective thicknesses information. **b** The microscopic image of an as-prepared TFT device. **c** Photograph of the obtained TFTs with Al_2_O_3_/HfO_2_ insulator on the flexible substrate

In this study, all the as-prepared TFTs with the bottom-gate top-contact configurations were fabricated by the typical photolithographic processes. The acceptable processing temperatures for obtaining an ideal insulator were discussed firstly. To study the area capacitance and leakage current density, the Al_2_O_3_ and HfO_2_ insulators prepared at different temperatures were measured from the Au/insulator/Au structure (Fig. S3). As shown in Fig. S4–S6, the Al_2_O_3_ and HfO_2_ insulators fabricated at a relatively lower temperature at 150 °C exhibited promising insulating properties and capacitance. Inspired by the utilization of nanolaminate structure to achieve the highly dense, humidity/oxygen-resistant, and flexible thin films such as Al_2_O_3_/MgO [31], Al_2_O_3_/TiO_2_ [38], and Al_2_O_3_/ZrO_2_ [39], the laminated Al_2_O_3_/HfO_2_ insulator with different layered numbers (Fig. S7) was fabricated here by controlling the ALD processing steps. The area capacitance and leakage current density of the different laminated Al_2_O_3_/HfO_2_ insulators were further investigated (Fig. S8) The five layers laminated Al_2_O_3_/HfO_2_ insulators showed the optimized insulating performance and reliability, which were labeled as Al_2_O_3_/HfO_2_ nanolaminates and used for the TFTs preparation. As shown in Fig. 1a, the typical photolithographic processes were applied for preparing the PI-based flexible TFTs with the Al_2_O_3_/HfO_2_ nanolaminates. The detailed procedures and parameters for each layer fabrication were depicted in the experimental section. The optical microscopic image of the as-prepared TFT device is given in Fig. 1b depicting the device with an effective channel length and width of 20 and 100 μm, respectively. After peeling off the PI substrate from the wafer, the as-fabricated PI-based flexible TFTs on a bending surface with a bending radius of 40 mm is displayed in Fig. 1c.

**Fig. 2:**
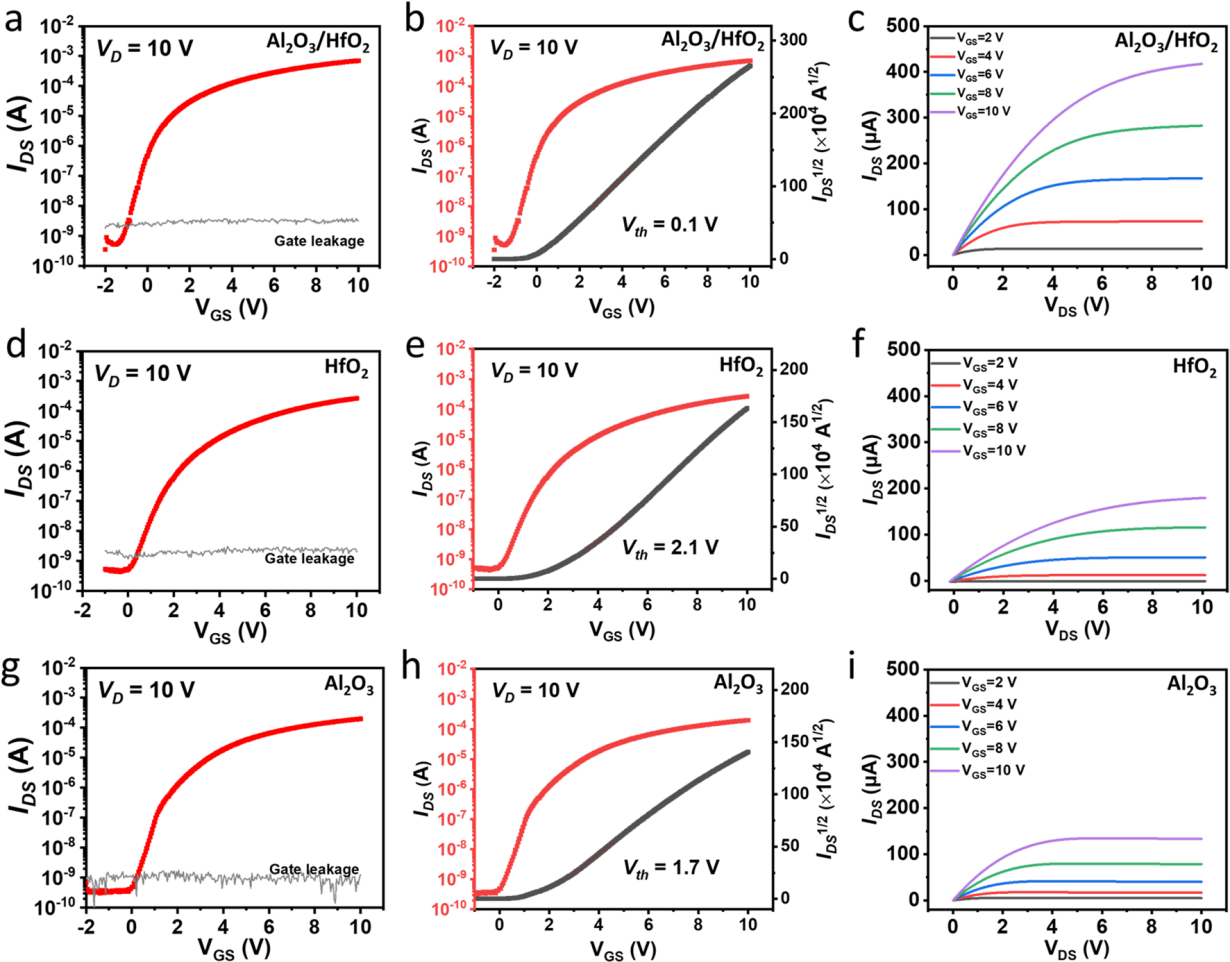
Transfer characteristics, Sqrt (*I*_DS_) curves, and output performances of the IGZO TFTs on the Si wafer with different insulators of **a–c** laminated Al_2_O_3_/HfO_2_, **d–f** HfO_2_, and **g–i** Al_2_O_3_ prepared by ALD at 150 °C

To demonstrate IGZO-based TFTs performances, comparisons were made with the 150 °C ALD-deposited insulators including Al_2_O_3_/HfO_2_ nanolaminates, HfO_2_, and Al_2_O_3_, and the electrical performance of the as-fabricated TFTs is measured and compared in Fig. 2, in which the *I*_DS_ (drain-to-source current), *I*_GS_ (gate leakage, drain-to-source current), and square-root-*I*_DS_ were plotted against the *V*_GS_ (gate-to-source voltage), and the *I*_DS_ was plotted against the *V*_DS_ (drain-to-source voltage). The thicknesses of the single layer HfO_2_ and Al_2_O_3_ were 4 nm. Figure 2a, d, and g shows the transfer characteristics of the TFTs with Al_2_O_3_/HfO_2_ nanolaminates, HfO_2_, and Al_2_O_3_ insulators, respectively, in which the V_DS_ was fixed at 10 V. The leakage current was displayed in the range of about 10^−9^ A for these three samples. The maximum on-current (*I*_on_) for the Al_2_O_3_/HfO_2_ nanolaminates based TFTs was as high as 0.70 mA, which was 350% and 260% higher than those with Al_2_O_3_ (0.20 mA) HfO_2_ (0.27 mA) insulators, respectively. As shown in Fig. 2b, e and h, the threshold voltage (*V*_th_) of 0.1, 2.1, and 1.7 V for Al_2_O_3_/HfO_2_ nanolaminates, HfO_2_, and Al_2_O_3_-based TFTs was obtained from the intersection points of the linear fitted square-root-I_DS_ against *V*_GS_ respectively. Figure 2c, f and I shows the output characteristics of Al_2_O_3_/HfO_2_ nanolaminates, HfO_2_, and Al_2_O_3_-based TFTs with the applied V_GS_ from 2 to 10 V with the increasing steps of 2 V. The saturation current and pinch-off ranges of these three samples were clearly observed, indicating that the channel current could be well controlled by the *V*_GS_. In addition, no current crowding behaviors for these three TFTs indicated good contact at the source/drain electrodes and channel interface. The *I*_DS_ of the TFTs with Al_2_O_3_/HfO_2_ nanolaminates (0.42 mA) at the *V*_GS_ of 10 V was at least twice larger than that with HfO_2_ (0.18 mA) and Al_2_O_3_ (0.13 mA) insulators, which could be attributed to the thermodynamical stability, high density, and good corrosion-resistance of Al_2_O_3_/HfO_2_ nanolaminates and higher saturation carriers mobility [40]. The effective mobility (*μ*), subthreshold swing (*SS*), transconductance (*g*_*m*_), and field-effect mobility (*μ*_FE_) can be calculated by the following equations [40–42]:

1 $$ I_{{\text{D}}} = \left( {\frac{W}{2L}C_{i} \mu } \right)\left( {V_{{{\text{GS}}}} - V_{{{\text{th}}}} } \right)^{2} $$

2 $$ SS = \frac{{dV_{{{\text{GS}}}} }}{{d\left( {\log I_{D} } \right)}} $$

3 $$ g_{m} = \frac{W}{L}\mu C_{i} V_{{{\text{DS}}}} $$

4 $$ \mu_{{{\text{FE}}}} = \frac{{Lg_{m} }}{{WC_{i} V_{{{\text{DS}}}} \left( {1 + \frac{{V_{E} - V_{{{\text{th}}}} }}{\mu }\frac{{{\text{d}}\mu }}{{{\text{d}}V_{{{\text{GS}}}} }}} \right) }} $$

where *L* and *W* are the length and width of the channel, *C*_*i*_ is the measured capacitance density (extracted from Fig.S2, S3, and S6 at the frequency of 100 kHz) of the insulator, *I*_D_, *V*_GS_, and *V*_th_ are the drain-to-source current, gate-to-source voltage, and threshold voltage, respectively. The calculated effective mobility *μ* for the TFTs with Al_2_O_3_/HfO_2_ nanolaminates, HfO_2_, and Al_2_O_3_ insulators was 9.7, 4.6, and 3.8 cm^2^ V^−1^ s^−1^, respectively. The calculated field-effect mobility for the TFTs with Al_2_O_3_/HfO_2_ nanolaminates was 8.2 cm^2^ V^−1^ s^−1^. Figure S9 shows the *V*_GS_ against log-scale I_DS_ plots of the Al_2_O_3_/HfO_2_ nanolaminates, HfO_2_, and Al_2_O_3_ insulators based TFTs. The *SS* for the TFTs with the Al_2_O_3_/HfO_2_ nanolaminates, HfO_2_, and Al_2_O_3_ insulators was 256, 482, and 389 mV dec^−1^. The performance parameters including μ, *I*_ON_/*I*_OFF_ ratio, *V*_th_, and SS at *V*_DS_ = 10 V of the TFTs are summarized in Table S1. In addition, the saturation currents per unit channel width of IGZO-based TFTs with different insulators are compared in Table 1. These results clearly indicated that the Al_2_O_3_/HfO_2_ nanolaminates deposited using the low-temperature ALD have delivered a superior performance of the TFTs. Compared with other nanolaminates such as ZrO_2_/Al_2_O_3_ (250 °C ALD) and Al_2_O_3_/MgO (70 °C ALD) [31, 43], the TFTs with Al_2_O_3_/HfO_2_ nanolaminates exhibited promising saturation current which could be applied for driving the high-power electronic components.

**Table 1:** Comparison of the saturation current per unit channel width of IGZO-based TFTs with different insulators

Channel length/width (μm)	Insulators materials	*V*_GS_ (V)	*V*_DS_ (V)	Saturated current per unit width (μA μm^−1^)	Refs.
60/1500	HfO_x_N_y_/HfO_2_/HfO_x_N_y_	5	5	0.2	[46]
300/1000	Nd: Al_2_O_3_	10	10.1	0.06	[47]
30/100	Al_2_O_3_	1	0.5	0.04	[48]
100/1000	ZrO_2_/HfO_2_	20	20	1.2	[40]
10/20	AlO_x_:Nd	10	5	0.5	[9]
50/500	Al_2_O_3_/MgO	10	10.1	1	[31]
10/50	SiO_x_	30	10.1	0.4	[36]
100/1500	Al_2_O_3_/TiO_2_	20	20	0.015	[38]
100/1000	SiO_2_	20	5	0.1	[44]
7/50	YAlO_x_	3	0.5	0.3	[30]
200/1000	HfGdO_x_	4	10	0.1	[50]
50/500	Annealed Al_2_O_3_	3	2	0.7	[51]
100/1000	Y_2_O_3_	30	20	0.1	[52]
300/1000	SiO_2_	30	5	0.3	[53]
250/1000	SiO_2_	40	40	0.25	[54]
100/150	HfO_2_	8	14	5.9	[55]
20/100	Al_2_O_3_/HfO_2_ nanolaminates	10	10	7.03	This work

**Fig. 3:**
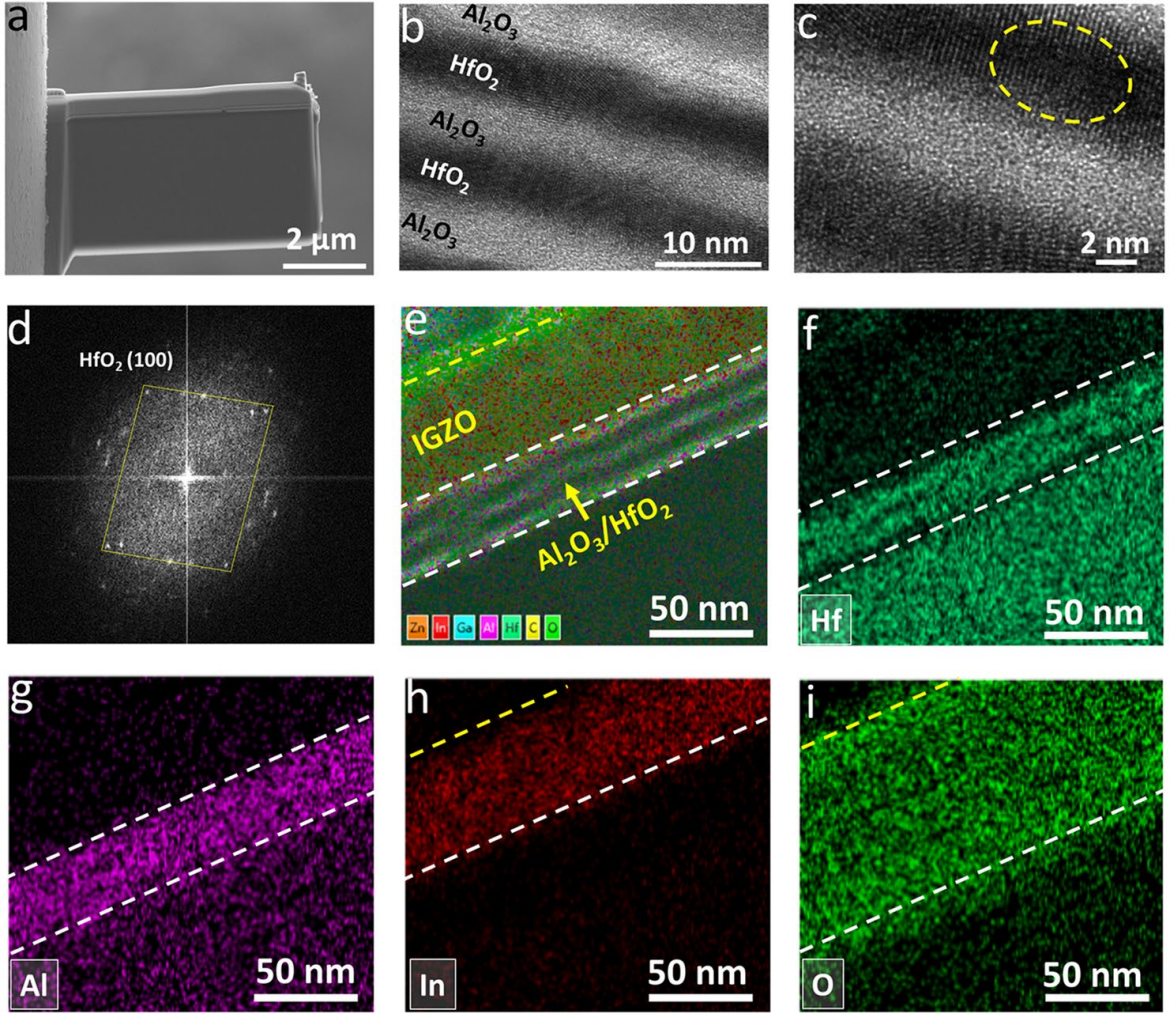
**a** SEM image of the TEM specimen prepared by FIB. **b** Cross-sectional TEM and **c** HRTEM images of the laminated Al_2_O_3_/HfO_2_ insulator prepared via ALD at 150 °C. **d** The fast fourier transform (FFT) diffraction pattern image obtained from the dotted area in panel **c**. **e** Cross-sectional TEM-EDS elemental mapping of elements Zn, In, Ga, Al, Hf, C, and O. EDS mapping of elements **f** Hf, **g** Al, **h** In, and **i** O

To characterize the morphology and microstructure of the Al_2_O_3_/HfO_2_ nanolaminates deposited via ALD at 150 °C, the cross-sectional TEM-EDS analysis was carried out on the IGZO/Al_2_O_3_/HfO_2_ layers of an actual TFTs device. As shown in Fig. S10, the TEM specimen was firstly prepared by a dual-beam FIB system. The SEM image of the obtained FIB specimen was given in Fig. 3a. Figure 3b shows the cross-sectional TEM image of the Al_2_O_3_/HfO_2_ nanolaminates. Five layers of the light (Al_2_O_3_) and dark (HfO_2_) stacking structure with the total thickness of ~ 20 nm were well observed with minimal interfacial roughness and good thickness uniformity. As exhibited from the HRTEM image in Fig. 3c, the Al_2_O_3_ layers were found to be amorphous, whereas the lattice fringe of HfO_2_ can be clearly observed indicating its crystalline state. In addition, a fuzzy interface between the crystallized HfO_2_ and amorphous Al_2_O_3_ was formed, which might be attributed to the formation of the aluminate phase at the laminated interface (similar to Al–Mg-O aluminate phase) [31]. The FFT diffraction pattern in Fig. 3d evidently showed the single-crystalline nature of HfO_2_ layers. The cross-sectional EDS mapping taken from part of the IGZO/Al_2_O_3_/HfO_2_ actual TFTs displayed the distribution of Zn, In, Ga, Al, Hf, C, and O elements (Fig. 3e). Figure 3f–i exhibited the EDS maps of the Hf, Al, In, and O elements, respectively. The sublayer structure of the Al_2_O_3_/HfO_2_ nanolaminates and sharper interface between the IGZO channel and insulator are found in Fig. 3f, h.

**Fig. 4:**
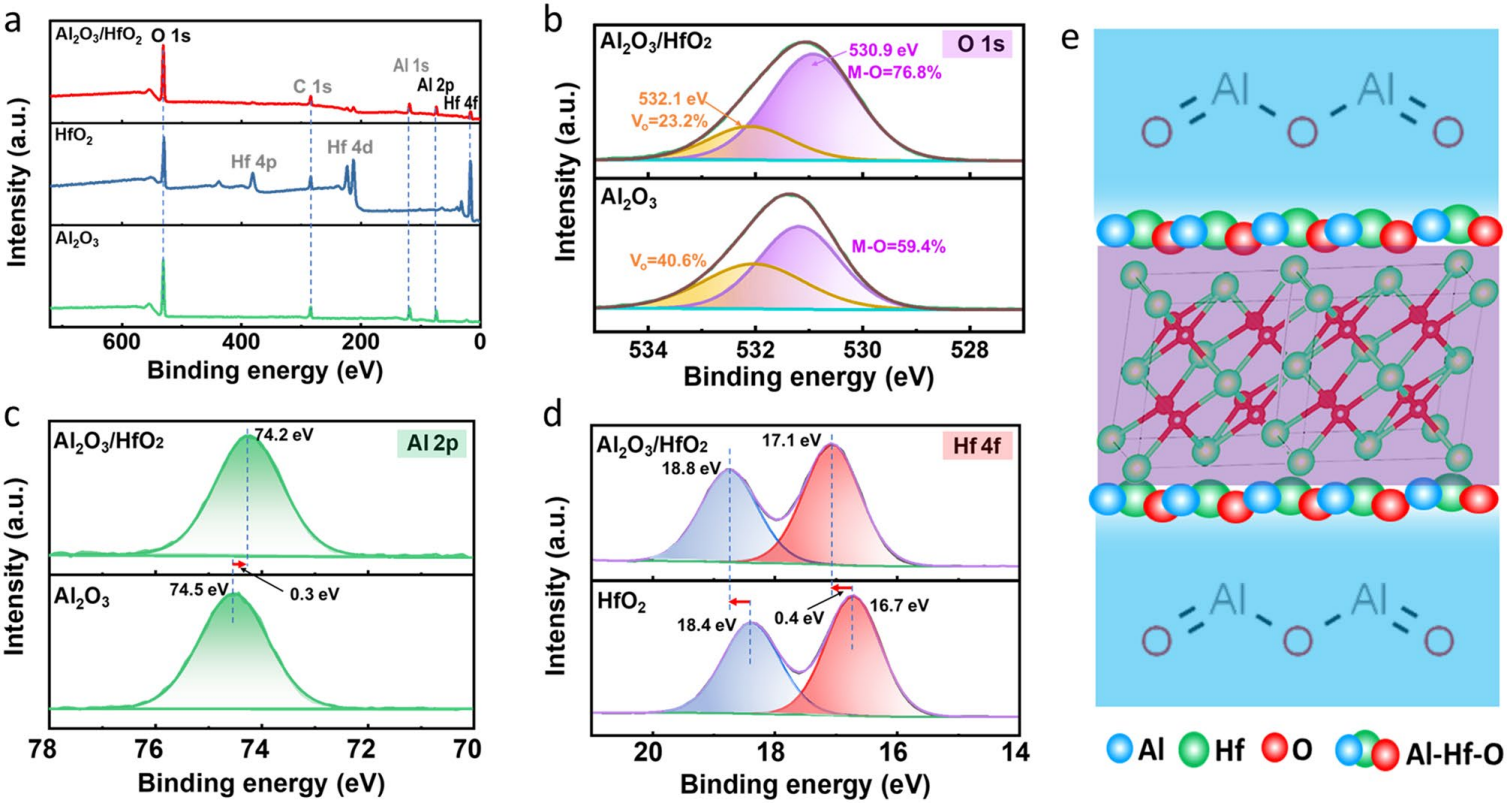
**a** X-ray photoelectron spectra of the Al_2_O_3_/HfO_2_, HfO_2_, and Al_2_O_3_ insulators prepared by ALD at 150 °C. High-resolution **b** O 1*s* and **c** Al 2*p* spectra of the Al_2_O_3_/HfO_2_ nanolaminates and Al_2_O_3_. **d** High-resolution Hf 4*f* spectra of the Al_2_O_3_/HfO_2_ nanolaminates and HfO_2_. **e** Schematic of the Al_2_O_3_/HfO_2_ nanolaminates with amorphous Al_2_O_3_, crystallized HfO_2_, and the aluminate (Al–Mg–O) phase at the interface

XPS spectra were also measured to investigate the elemental composition and chemical states of the Al_2_O_3_/HfO_2_ nanolaminates, HfO_2_, and Al_2_O_3_ insulators. As shown in Fig. 4a, the peaks corresponding to C, O, Al, and Hf elements were found in the Al_2_O_3_/HfO_2_ nanolaminates XPS spectrum. The peaks belonging to C, O, and Hf elements and peaks corresponding to C, O, and Al elements were observed in the HfO_2_ and Al_2_O_3_ XPS spectra, respectively. The O 1* s* XPS spectra of the Al_2_O_3_/HfO_2_ nanolaminates and Al_2_O_3_ are shown in Fig. 4b and fitted by two peak components through Gaussian fitting. The peaks centered at high binding energy of 532.1 eV correspond to oxygen vacancy, whereas the peaks at 530.9 eV are related to oxide lattices (metal-oxide bonds here) [40]. The relative content of oxygen vacancy for Al_2_O_3_/HfO_2_ nanolaminates was found to be 23.2%, which was smaller than that of 40.6% for Al_2_O_3_. The Al_2_O_3_/HfO_2_ nanolaminates with low oxygen vacancy were beneficial for enhancing the TFTs performance [40, 44]. Figure 4c, d presents the Al 2*p* and Hf 4*f* core-level XPS spectra for the Al_2_O_3_/HfO_2_ nanolaminates, Al_2_O_3_, and HfO_2_. The core-level peaks of Al 2*p* and Hf 4*f* in Al_2_O_3_ and HfO_2_ were found at 74.5 eV (Al 2*p*), 18.4 eV (Hf 4*f*_5/2_), and 16.7 eV (Hf 4*f*_7/2_), in agreement with the binding energy in previous reports [45]. As for the Al_2_O_3_/HfO_2_ nanolaminates comparing with Al_2_O_3_ and HfO_2_, the Al 2*p* core-level peak was shifted toward lower binding energy of 74.5 eV, and Hf 4*f* core-level peaks were shifted toward higher binding energy of 18.8 eV (Hf 4f 5/2) and 17.1 eV (Hf 4*f*_7/2_). These binding energy shifts of Al 2*p* and Hf 4*f* in Al_2_O_3_/HfO_2_ nanolaminates are resulted from the difference in electronegativities of Al (1.61) and Hf (1.32) [31, 46]. Therefore, as schematically shown in Fig. 4e, the aluminate phase of Al–Hf–O was formed between the sublayers of Al_2_O_3_ and HfO_2_ in Al_2_O_3_/HfO_2_ nanolaminates. These aluminate phases with enhanced thermodynamical stability, high density, and good corrosion-resistance have been reported in similar laminated structures such as Al_2_O_3_/ZrO_2_, Al_2_O_3_/TiO_2_, and Al_2_O_3_/MgO [31, 38, 39]. As a result, this Al–Hf–O chemical bonding improved the reliability of the laminated Al_2_O_3_/HfO_2_ insulator. Therefore, the Al_2_O_3_/HfO_2_ nanolaminates with the layered structure of amorphous Al_2_O_3_, the aluminate phase in sublayers, and crystallized HfO_2_ could be applied as an ideal dielectric for the high-performance TFTs.

**Fig. 5:**
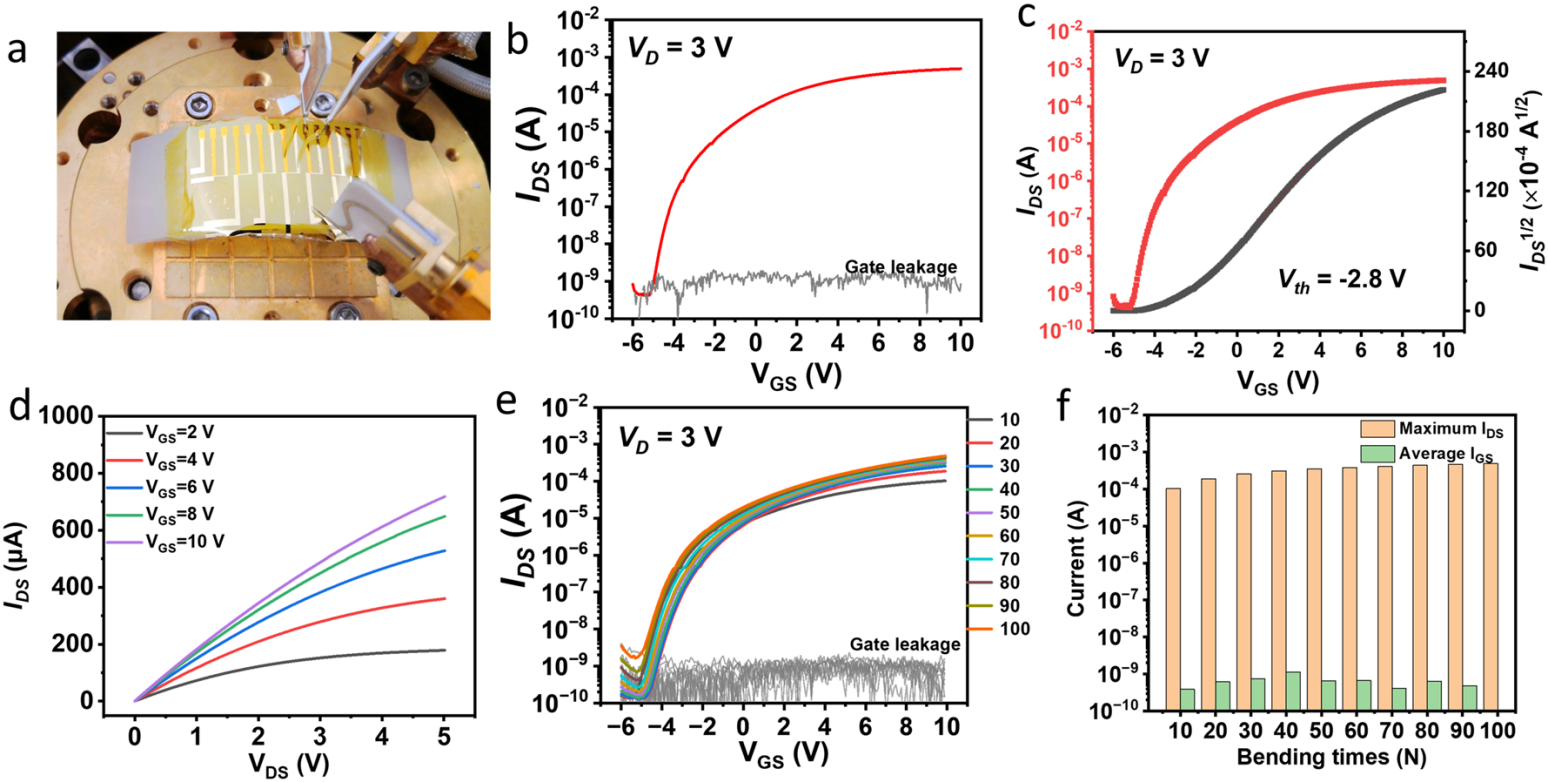
**a, g** Photograph of the PI-based flexible TFTs with lAl_2_O_3_/HfO_2_ nanolaminates tested on a bending surface with a bending radius of 40 mm. **b** Transfer characteristics, **c** Sqrt (I_DS_) curve, and **d** output performances of the flexible TFTs tested at a bending radius of 40 mm. **e** Transfer characteristics and **f** the maximum IDS and average IGS of the flexible IGZO-based TFTs after repeated bending for 100 times at the bending radius of 40 mm

The flexible TFTs on the PI substrate were fabricated with 150 °C ALD-deposited Al_2_O_3_/HfO_2_ nanolaminates. As shown in Fig. 5a, the PI-based flexible TFTs with Al_2_O_3_/HfO_2_ nanolaminates could be tested on a bending surface with a bending radius of 40 mm. Figure 5b presents the transfer characteristics of the PI-based flexible TFTs with the fixed *V*_DS_ of 3 V. The leakage current was in the range of ~ 10^−9^ A. The maximum *I*_on_ for PI-based flexible TFTs was up to as 0.83 mA. The obtained *I*_ON_/*I*_OFF_ ratio was higher than 10^6^. The *V*_th_ of − 2.8 V was extracted from the intersection point of the linear fitted square-root-I_DS_ against *V*_GS_ (Fig. 5c). Figure 5d shows the output characteristics of Al_2_O_3_/HfO_2_ nanolaminates-based flexible TFTs with the applied *V*_GS_ from 2 to 10 V with the increasing steps of 2 V. The saturation I_DS_ could be observed from the output curves. The *I*_DS_ of this flexible TFTs was up to 0.72 mA at the *V*_GS_ of 10 V. In addition, the *V*_GS_ against log-scale *I*_DS_ curves of the flexible TFTs are also plotted in Fig. S11. Based on Eq. (2), the *SS* of this flexible TFTs with the Al_2_O_3_/HfO_2_ nanolaminates was calculated as 319 mV dec^−1^. Compared with other PI-based organic or inorganic flexible TFTs [30, 37], the flexible TFTs with Al_2_O_3_/HfO_2_ nanolaminates showed promising performance including the carriers mobility and output current. Transfer characteristics of the fabricated flexible IGZO thin-film transistors after repeated bending for 100 times at the bending radius of 40 mm are shown in Fig. 5e. Furthermore, the stable maximum *I*_DS_ and average gate leakage of the as-prepared TFTs are also summarized in Fig. 5f. These satisfactory electrical performances of the flexible IGZO-based TFTs exhibited their promising flexibility and endurability.

**Fig. 6:**
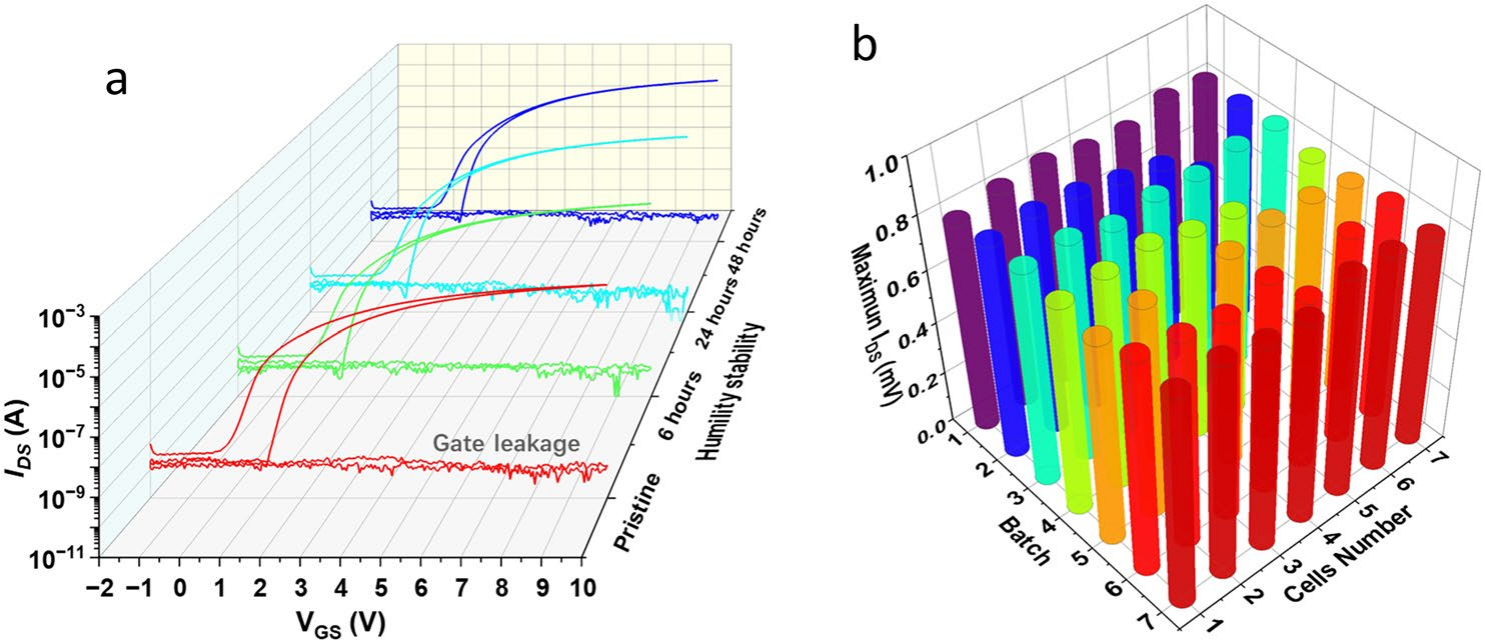
**a** Transfer characteristics and hysteresis behavior of the IGZO-based TFTs with nanolaminate Al_2_O_3_/HfO_2_ insulator in a pristine state and after being stored in a laboratory environment (relative humidity of 60– 70%, temperature of 25–30 °C) for 6, 24, and 48 h. **b** 3D bars map showing maximum *I*_DS_ for 7 batch (each batch has seven cells) IGZO-based TFTs

The humidity stability and hysteresis behavior of the as-prepared IGZO-based TFTs with nanolaminate Al_2_O_3_/HfO_2_ insulator were conducted by storing the devices in a laboratory environment (relative humidity of 60–70%, temperature of 25–30 °C) at different times. As shown in Fig. 6a, the gate leakage of IGZO-based TFTs with nanolaminate Al_2_O_3_/HfO_2_ insulator is kept stable and at a relatively low value of about 10^–10^ A. Meanwhile, the TFTs exhibited ideal transfer behaviors with small hysteresis after exposure to a relative humidity of 60–70%, and a temperature of 25–30 °C environment for 48 h. The reliability of the set of IGZO-based TFTs with nanolaminate Al_2_O_3_/HfO_2_ insulator was also processed (Fig. 6b). Seven batches (each batch has seven cells) total of 49 IGZO-based TFT cells were tested. As shown in Fig. 6b, two cells in batch 3 and batch 4 were damaged. The other 47 cells worked well with an average maximum *I*_DS_ of 0.79 mV. The yield of IGZO-based TFTs with nanolaminate Al_2_O_3_/HfO_2_ insulator reaches 95%.

## Conclusions

Flexible TFTs on PI substrates were successfully fabricated using the Al_2_O_3_/HfO_2_ nanolaminate which was deposited by ALD at 150 °C. The Al_2_O_3_/HfO_2_ nanolaminate was demonstrated with the layered structure of amorphous Al_2_O_3_, crystallized HfO_2_, and the aluminate Al–Hf–O phase at sublayers interface. The as-prepared TFTs without any post-annealing presented the carrier mobility of 9.7 cm^2^ V^−1^ s^−1^, ON/OFF ratio ~ 1.3 × 10^6^, subthreshold voltage of 0.1 V, saturated current up to 0.83 mA, and subthreshold swing of 0.256 V dec^−1^, as well as withstand the bending radius of 40 mm. The as-prepared TFTs possess satisfactory humidity stability in a relative humidity of 60–70%, a temperature of 25–30 °C environment. The yield of IGZO-based TFTs with the nanolaminate insulator reaches 95%. We believe this Al_2_O_3_/HfO_2_ nanolaminate could be one of the ideal candidates for high-performance electronics.

## Supplementary Information

Below is the link to the electronic supplementary material.

Supplementary file1 (PDF 934 KB)
