# Supplementary material for: Al2O3/HfO2 Nanolaminate Dielectric Boosting IGZO-Based Flexible Thin-Film Transistors
Source: Nanomicro Lett. 2022 Sep 27;14:195. doi: 10.1007/s40820-022-00929-y (PMC9515270; doi:10.1007/s40820-022-00929-y)
Supplement: Supplementary file 1 — Supplementary file1 (PDF 934 KB) [file 40820_2022_929_MOESM1_ESM.pdf]

Supporting Information for

Al_2O_3/HfO_2 Nanolaminate Dielectric Boosting IGZO-Based Flexible Thin-Film Transistors

Qiuwei Shi[1, 2, #], Izzat Aziz[1, #], Jin-Hao Ciou[1], Jiangxin Wang[1], Dace Gao[1], Jiaqing Xiong[1] and Pooi See Lee[1, *]

[1]School of Materials Science and Engineering, Nanyang Technological University 50 Nanyang Avenue, Singapore 639798, Singapore

[2]School of Chemistry and Materials Science, Nanjing University of Information Science and Technology, Nanjing 210044, P. R. China

[#]Qiuwei Shi and Izzat Aziz contributed equally to this work.

*Corresponding author. E-mail: pslee@ntu.edu.sg (P. S. Lee)

Supplementary Figures and Tables

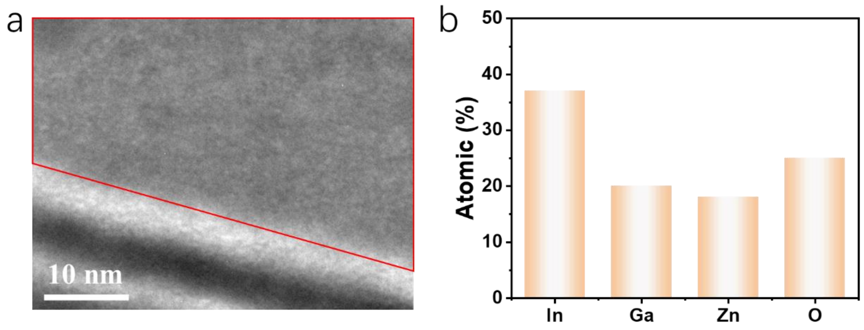

Fig. S1 (**a**) Cross-sectional FIB-TEM image of the amorphous IGZO (in red wireframe) active layer. (**b**) EDX result of the atomic percentage of the IGZO active layer

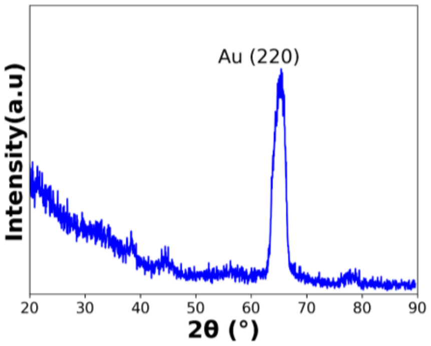

Fig. S2 (**a**) XRD spectra of the IGZO active layer on Au substrate. It is demonstrated that the as-prepared IGZO is in an amorphous state

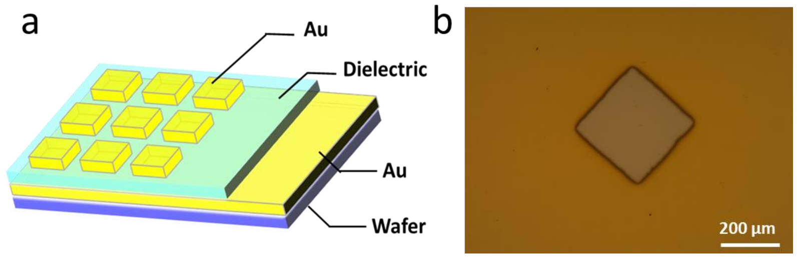

Fig. S3 (**a**) Schematic of the Au/insulator (Al_2O_3 or HfO_2)/Au metal-insulator-metal structure for leakage current density and capacitance measurement. (**b**) The microscope image of the top Au electrode with the size of 300 μm × 300 μm

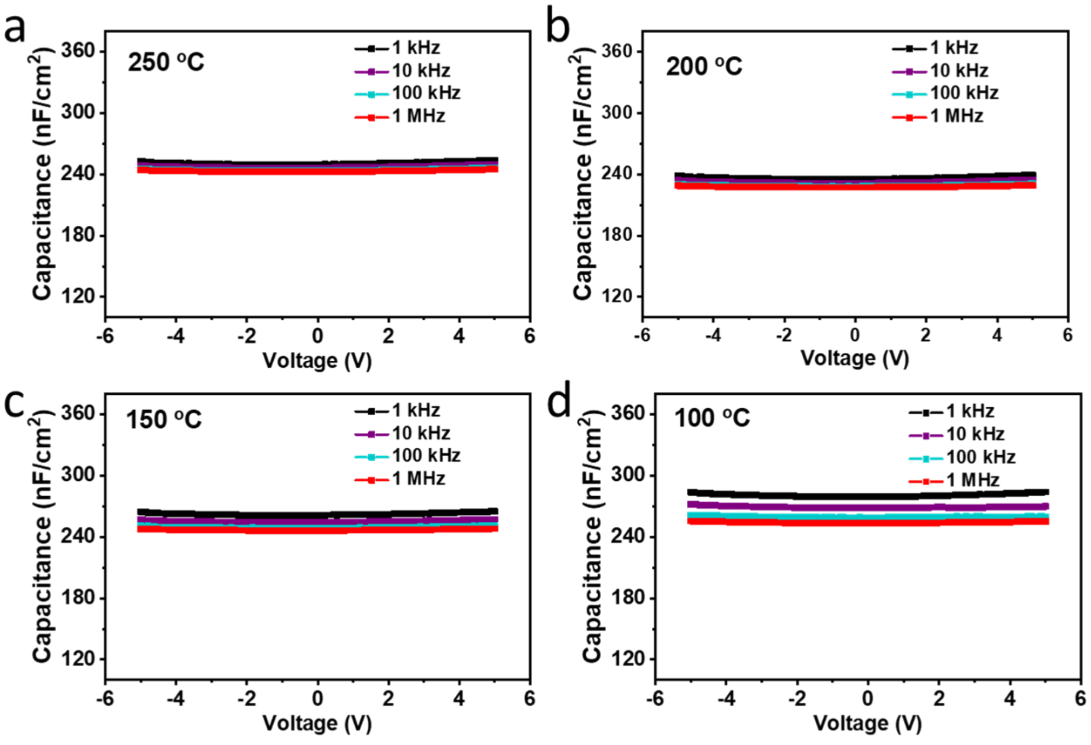

Fig. S4 Capacitance vs voltage measurement of the 20 nm Al_2O_3 insulators prepared with different ALD temperature of (**a**) 250, (**b**) 200, (**c**) 150 and (**d**) 100 °C at the various frequency of 1 kHz, 10 kHz, 100 kHz and 1 MHz

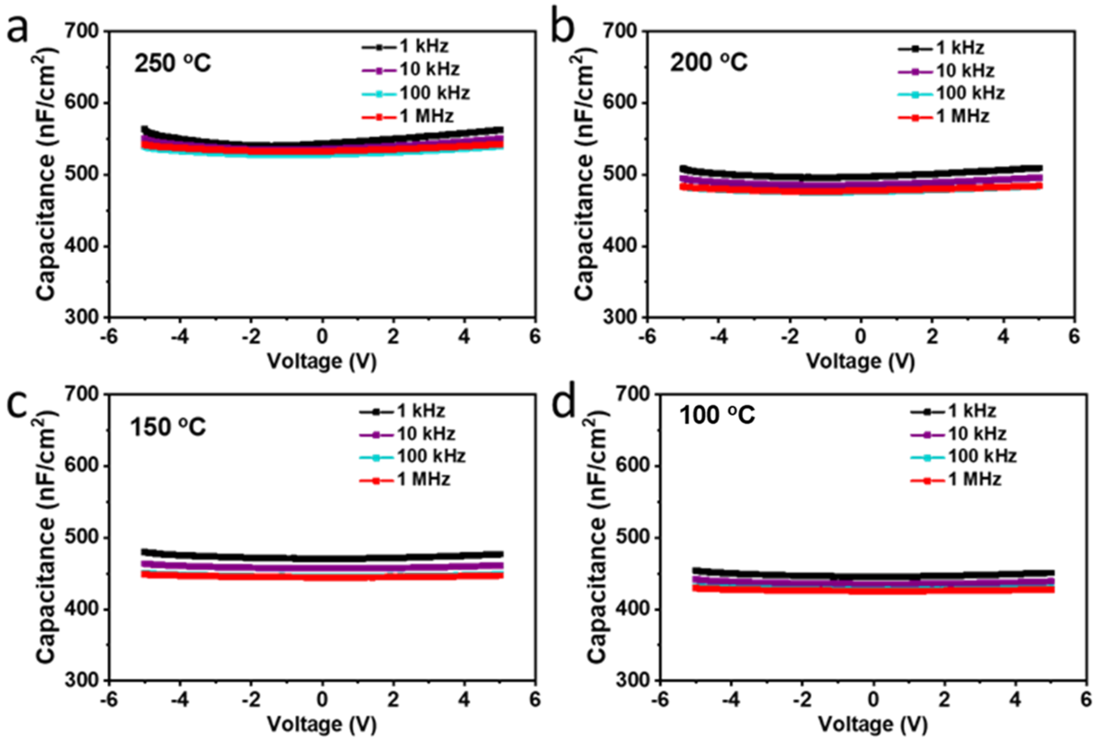

Fig. S5 Capacitance vs voltage measurement of the 20 nm HfO_2 insulators prepared with different ALD temperature of (**a**) 250, (**b**) 200, (**c**) 150 and (**d**) 100 °C at the various frequency of 1 kHz, 10 kHz, 100 kHz and 1 MHz

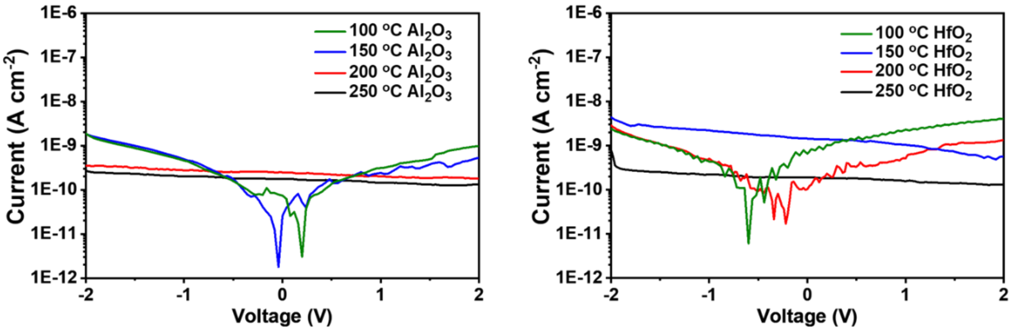

Fig. S6 (**d**) Comparison of the leakage current density of the 20 nm (**a**) Al_2O_3 and (**b**) HfO_2 insulators prepared with different ALD temperature of (**a**) 250, (**b**) 200, (**c**) 150 and (**d**) 100 °C

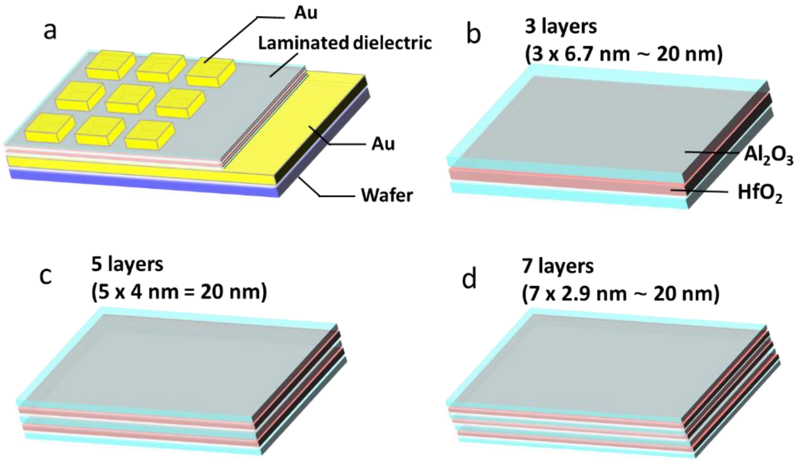

Fig. S7 (**a**) Schematic of the Au/nanolaminated dielectric/Au structure for leakage current density and capacitance measurement. Schematic of the 20 nm total thick nanolaminated Al_2O_3/HfO_2 insulators with different layer numbers of (**b**) 3 layers, (**c**) 5 layers, and (**d**) 7 layers

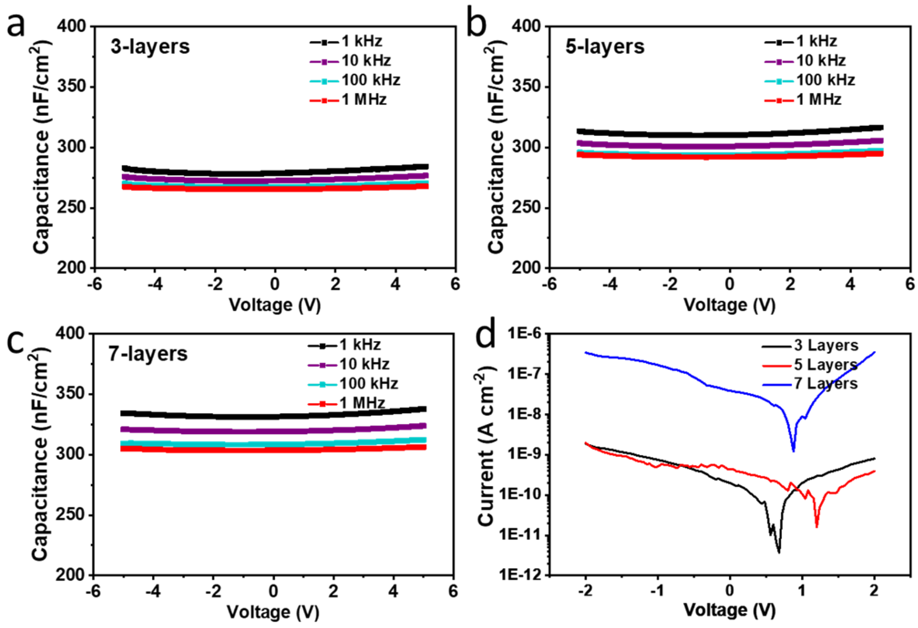

Fig. S8 (**a–c**) Capacitance vs voltage measurement of the as-prepared 20 nm nanolaminated Al_2O_3/HfO_2 insulators prepared at an ALD temperature of 150 °C. (**d**) Comparison of the leakage current density of the 3, 5 and 7 layered nanolaminated Al_2O_3/HfO_2 insulators

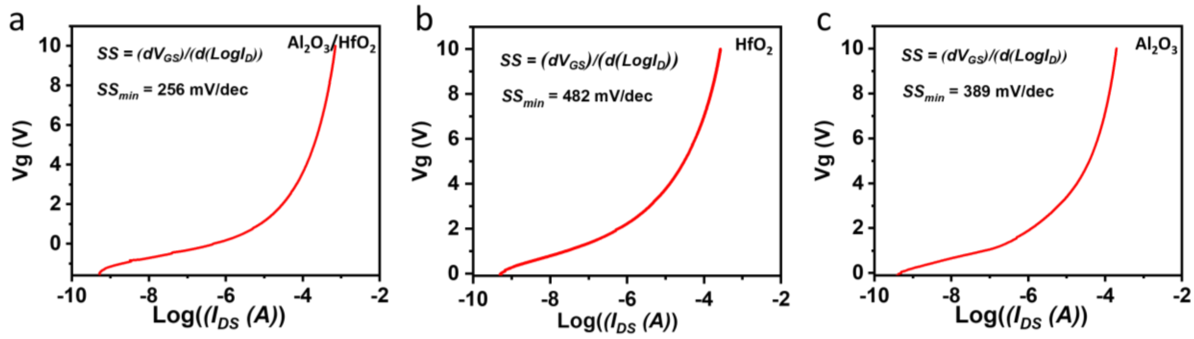

Fig. S9 V_{GS} against log-scale I_{DS} plots of the (**a**) laminated Al_2O_3/HfO_2, (**b**) HfO_2, and (**c**) Al_2O_3 insulators based TFTs

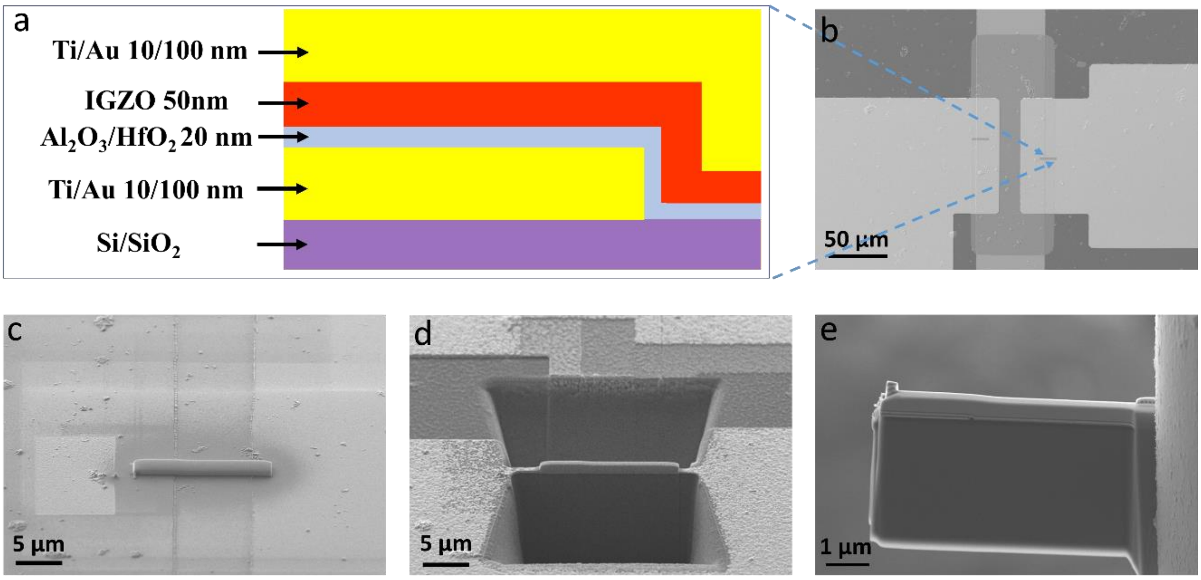

Fig. S10 TEM specimen preparation via focus-ion-beam (FIB). (**a**) Schematic of the layered structure and (**b** and **c**) SEM images of the FIB operation area of the TFTs with the laminated Al_2O_3/HfO_2 insulator. (**d**) The thinning and cutting process via FIB for the specimen (**e**) SEM image of the prepared TEM specimen after FIB lift-out process

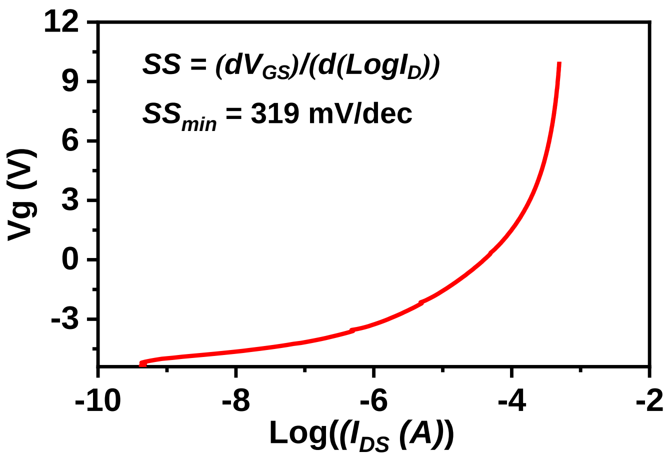

Fig. S11 V_{GS} against log-scale I_{DS} plots of the PI-based TFTs with the laminated Al_2O_3/HfO_2 insulator

Table S1 Performance parameters including μ, I_{ON}/I_{OFF} ratio, V_{th}, and SS at V_{DS} = 10 V of the TFTs with different insulators of laminated Al_2O_3/HfO_2, Al_2O_3, and HfO_2

**Insulator materials**	**Mobility (μ, cm^2/V s)**	**ON/OFF ratio**	**Subthreshold Voltage (V_{th})**	**Subthreshold swing (SS, mV dec^{-1})**
Laminated Al_3O_3/HfO_2	9.7	~1.3×10^6	0.1	256
Al_3O_3	4.6	~5.3×10^5	1.7	389
HfO_2	3.8	~5.5×10^5	2.1	482
